# Barrier films for the prevention of acute radiation dermatitis in breast cancer: A systematic review and meta-analysis of randomised controlled trials

**DOI:** 10.1016/j.breast.2023.07.001

**Published:** 2023-07-05

**Authors:** Cas Stefaan Dejonckheere, Egon Dejonckheere, Julian Philipp Layer, Katharina Layer, Gustavo Renato Sarria, David Koch, Alina Abramian, Christina Kaiser, Kira Lindner, Anne Bachmann, Teresa Anzböck, Fred Röhner, Frederic Carsten Schmeel, Leonard Christopher Schmeel

**Affiliations:** a Department of Radiation Oncology, University Hospital Bonn, 53127, Bonn, Germany; b Faculty of Psychology and Educational Sciences, KU Leuven, 3000, Leuven, Belgium; c Department of Medical and Clinical Psychology, Tilburg School of Social and Behavioural Sciences, 5037, Tilburg, the Netherlands; d Institute of Experimental Oncology, University Hospital Bonn, 53127, Bonn, Germany; e Department of Gynaecology, Division of Senology, University Hospital Bonn, 53127 Bonn, Germany; f Department of Gynaecology, Division of Gynaecological Oncology, University Hospital Bonn, 53127, Bonn, Germany; g Department of Neuroradiology, University Hospital Bonn, 53127, Bonn, Germany

**Keywords:** Acute radiation dermatitis, Breast cancer, Radiotherapy, Prevention, Barrier film, Hydrofilm, Mepitel film, Systematic review, Meta-analysis

## Abstract

**Purpose:**

Radiation dermatitis (RD) is the most common side effect of adjuvant whole-breast or chest wall irradiation, majorly impacting quality of life in numerous patients. The use of barrier films (polyurethane dressings such as Hydrofilm® and Mepitel® film remaining on the skin for the duration of the radiation treatment) has been investigated as a prophylactic measure in several prospective trials. Here, we critically appraise the available evidence behind preventive barrier film application in the context of breast cancer treatment.

**Methods:**

International literature was reviewed and high-quality randomised controlled trials (RCTs) were included in this meta-analysis.

**Results:**

The results of 5 RCTs (663 patients; >90% Caucasian) were analysed. Overall, barrier films lead to improved clinician- and patient-reported outcomes: fewer grade ≥2 RD (11% vs. 42%; OR = 0.16; *p* < 0.001) and moist desquamation (2% vs. 16%; OR = 0.12; *p* = 0.006), as well as less patient-reported pain (standardised mean difference [SMD] −0.51; *p* < 0.001), itching (SMD −0.52; *p* = 0.001), burning (SMD −0.41; *p* = 0.011), and limitations in daily activities (SMD −0.20; *p* = 0.007). Furthermore, barrier films have a high acceptance rate among patients, as well as a favourable cost-benefit ratio. Possible side effects due to its application are mild and mostly self-limiting. Overall, there was a lack of information on the radiation treatment techniques used.

**Conclusion:**

The evidence presented in this meta-analysis suggests that barrier films are an excellent tool in the prevention of RD among Caucasian patients receiving whole-breast or chest wall irradiation. Its use should therefore be considered routinely in these patients.

## Highlights

• Meta-analysis of 5 trials investigating Hydrofilm® and Mepitel® barrier films.
• Reduction of incidence and severity of radiation dermatitis and moist desquamation.
• Fewer patient-reported pain, itching, burning, and limitations in daily activities.
• Mild and self-limiting side effects, high acceptance, favourable cost-benefit ratio.
• Mainly Caucasian patients and limited information on radiation treatment techniques.

## 1 Introduction

Radiation dermatitis (RD) remains the most common acute side effect of adjuvant whole-breast or chest wall irradiation [1,2]. Its pathophysiology is complex, but disruptions of the skin barrier function through direct DNA damage to the epidermal basal layer cells play a central role. The main extrinsic risk factor for RD is the skin dose. Recent advances in dose-fractionation regimens (e.g. hypofractionation causing fewer damage to healthy tissues without compromising tumour control) and radiation treatment techniques (e.g. higher photon energies, intensity-modulated radiation therapy [IMRT], and volumetric intensity-modulated arc therapy [VMAT]) can reduce this skin dose, resulting in fewer and milder RD. In particular, skin folds or areas of frequent arm movement (e.g. the axilla) are most susceptible to radiation-induced skin toxicities due to damage of the keratinised superficial skin layers from friction or maceration. Other extrinsic risk factors for RD in breast cancer irradiation are use of a bolus (leading to skin dose build-up) and boost administration. Proven intrinsic risk factors include large breast size (requiring a higher number of monitoring units, thus leading to increased inflammation), and darker skin complexion (darker skin types naturally tend to have a more rapid pigmentation tendency and accompanying inflammatory reaction) [[3], [4], [5], [6]]. In more severe cases involving moist desquamation, treatment interruptions might be necessary, possibly compromising disease control [7]. Furthermore, the development of RD impairs quality of life and self-image [[8], [9], [10]]. Topical corticosteroids are effective in reducing RD-related symptoms such as pain, burning, or itching, however, their widespread and prolonged use remains limited due to the associated side effect profile [7,11]. More importantly, topical corticosteroids should preferably be used on intact skin only, as they might otherwise even delay wound healing or promote infection when applied in the context of advanced moist desquamation. Other preventive and therapeutic options have been studied, often with conflicting results due to methodological differences, leading to substantial variation in RD management between practitioners and clinics [7,12,13].

Barrier films (e.g. Hydrofilm®, Mepitel® film) have been standard of care in wound management for several decades [14,15]. Both are sterile, transparent, and breathable polyurethane films, which differ in the adhesive used (Hydrofilm has a hypoallergenic polyacrylate, whereas Mepitel film uses a silicone-based adhesive). They can be applied directly on the skin (or wound) and protect it from friction and excess moisture by providing a semi-permeable mechanical barrier between the damaged basal skin layer and any potential additional trauma [16]. RD and especially moist desquamation have a predilection for areas prone to friction such as the axilla and inframammary fold. The use of barrier films as a protective layer in the context of breast or chest wall irradiation is therefore of major interest, as it facilitates repair of the radiation-damaged tissue while protecting against any additional trauma. Due to their clinically insignificant bolus effect and high patient tolerability (when showering or exercising and regardless of clothing), barrier films can remain on the skin for the entire duration of the radiation course, which promotes patient comfort [[17], [18], [19], [20]].

Initial trials sought to determine the therapeutic effect of barrier films, i.e. they were applied as soon as an RD-associated erythema became apparent [15,21]. Subsequent studies investigated the effect as a prophylactic measure. Herein, we review the data supporting the use of barrier films for the prevention of acute RD in the setting of adjuvant whole-breast or chest wall irradiation.

## 2 Materials and methods

**Fig. 1:**
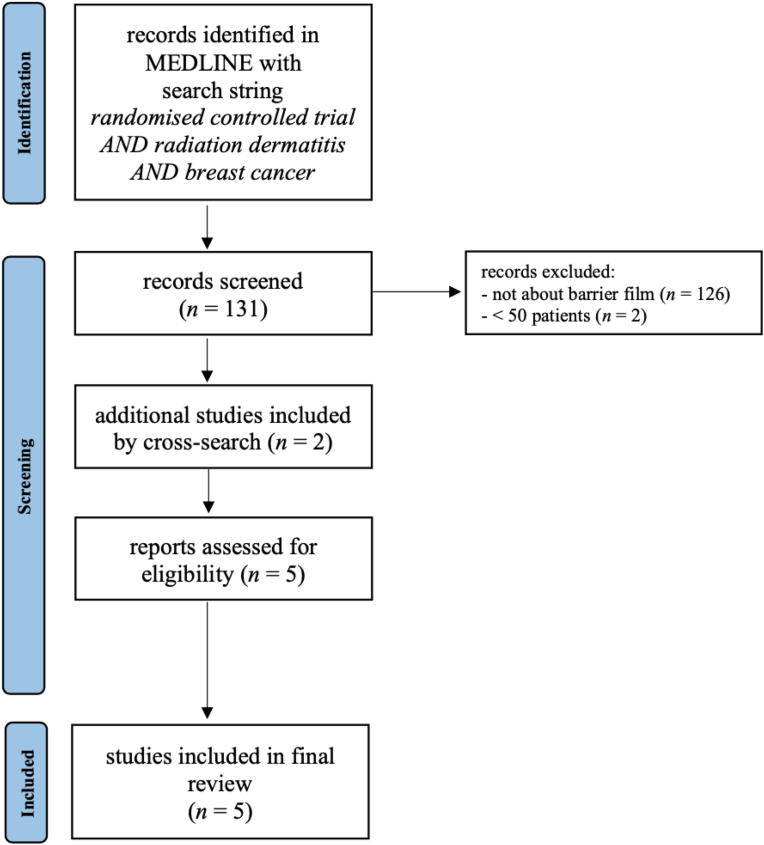
Preferred Reporting Items for Systematic Reviews and Meta-Analysis (PRISMA) flowchart of literature research and selection.

We conducted a comprehensive literature search of the MEDLINE database, using PubMed as the primary search engine. Studies published between January 1st, 1977 and March 1st, 2023 matching the search string *randomised controlled trial AND radiation dermatitis AND breast cancer* were screened for inclusion based on title and abstract. All randomised controlled trials (RCTs) that investigated the use of barrier films in at least 50 breast cancer patients undergoing irradiation were included. Studies reporting on barrier creams or film-forming gels were excluded from the analysis, as these products possess different properties which would increase the heterogeneity of the results of the intervention. Additional studies were identified by cross-searching the already included articles’ references. A flowchart of literature research and selection is shown in Fig. 1.

All included papers were independently appraised by 2 authors (C.S.D. and L.C.S) and data were collected from the manuscripts, supplements, and study protocols (where available). The individual risk of bias was assessed using the revised Cochrane tool for randomised trials [22]. The different domains were independently assessed by two reviewers and a traffic light plot was generated (Supplement 1). This study was designed in accordance with the Preferred Reporting Items for Systematic Reviews and Meta-Analysis (PRISMA) statement [23].

Mean, median, standard deviation (SD), and range were calculated for all applicable clinical data. For comparable outcomes between studies, the pooled effect size was estimated by calculating the odds ratio (OR) with a 95% confidence interval (CI), using a random effects model and results were summarised in a forest plot [24]. A *p*-value <0.05 was considered as statistically significant. Heterogeneity between studies was assessed by calculating I^2^ with cut-offs as defined by Higgins et al. [25]. The analysis was carried out using *R* version 4.1.2 (*R* Foundation for Statistical Computing, Vienna, Austria).

## 3 Results

### 3.1 Trial, patient, and radiation treatment characteristics

A total of 5 RCTs, reporting on 663 patients, was included in the final analysis [[17], [18], [19], [20],^26^]. Of these, 2 investigated Hydrofilm and 3 Mepitel film). Four trials used an intrapatient control, i.e. the irradiated breast or chest wall was divided into a medial and a lateral compartment, and each of these compartments was randomised to receive either barrier film or standard of care. Only the most recent trial used a 2:1 interpatient random allocation, based on the proposed superiority of Mepitel film demonstrated in the previous trials [20]. Table 1 depicts the general characteristics of the included RCTs.

**Table 1:** General characteristics of the included randomised controlled trials on the prevention of acute radiation dermatitis using barrier films (*n* = 5), including 663 patients.

authors	year	region (centers)	time frame	randomisation	intervention	control	*n*^a^	reference
Herst et al.	2014	New Zealand (1)	10/2012−04/2013	intrapatient	Mepitel film	aqueous cream	78	[16]
Schmeel et al. (a)	2018	Germany (1)	09/2016−09/2017	intrapatient	Hydrofilm	5% urea lotion	56	[17]
Møller et al.	2018	Denmark (3)	10/2015−04/2016	intrapatient	Mepitel film	moisturising lotion	79	[25]
Schmeel et al. (b)	2019	Germany (2)	03/2018−06/2019	intrapatient	Hydrofilm	5% urea lotion	74	[18]
Behroozian et al.	2022	Canada (3)	01/2020−05/2022	2:1	Mepitel film	aqueous cream	376	[19]

a Patients included in the statistical analyses of the respective trials.

All included trials recruited patients undergoing whole-breast irradiation following lumpectomy as well as patients receiving chest wall irradiation after mastectomy. Behroozian et al. only included lumpectomy patients if they were at a higher risk of developing acute radiation dermatitis (defined as postoperative bra size ≥36 inches of cup size ≥ C). Conventional fractionation (50 Gy in 25 fractions) and moderate hypofractionation (40−42.6 Gy in 15–16 fractions) were allowed, as well as boost administration (10−16 Gy in 5–8 fractions) and/or the use of a bolus to increase skin dose. If this was the case, patients were stratified accordingly to minimise confounding. Table 2 shows the individual and overall patient and radiation treatment characteristics.

**Table 2:** Patient and radiation treatment characteristics across selected trials.

authors	surgery		fractionation		boost (%)	bolus (%)
	lumpectomy (%)	mastectomy (%)	CF (%)	mHF (%)		
Herst et al.	56	44	50	50	36	8
Schmeel et al. (a)	100	0	100	0	31	0
Møller et al.	80	20	25	75	8	20
Schmeel et al. (b)	100	0	0	100	34	0
Behroozian et al.	58^a^	42	7	93	29	13
**total**	**79**	**21**	**36**	**64**	**27**	**8**

CF = conventional fractionation; mHF = moderate hypofractionation.

a In this trial, only patients at a higher risk of developing radiation dermatitis were included. Therefore, patients were only eligible after lumpectomy if they had a postoperative bra size ≥36 inches or cup size ≥ C.

### 3.2 Intervention properties and bolus effect

Two trials investigated the use of Hydrofilm, a polyurethane film dressing, while the other 3 trials used Mepitel film, a silicone-based polyurethane film. In all trials, barrier film was used from the first day of radiation treatment onwards and a moisturising cream or lotion was used as the standard of skin care control, a common recommendation which is considered good clinical practice [7,27].

Skin dose contributes to the risk of RD development. It is therefore of interest to determine the possible bolus effect of an applied barrier film [6,28]. All trials carried out phantom studies: with application of a barrier film, there is a negligible bolus effect of 0.12 mm, resulting in a clinically insignificant skin dose build-up. Barrier films can thus safely remain on the skin for the entire radiation course, which was the case in all trials.

### 3.3 Clinician-reported outcome

Clinician-reported outcome has long been regarded as the gold standard for RD assessment [20,29]. Commonly used scoring tools include the Radiation Therapy Oncology Group (RTOG) and the Common Terminology Criteria for Adverse Events (CTCAE) scales, which evaluate skin toxicity on a scale from 0 (no symptoms) to 5 (death) [30,31]. Furthermore, the Radiation-Induced Skin Reaction Assessment Scale (RISRAS), a validated tool to assess the visible extent of skin reaction, can be used by clinicians to evaluate erythema, dry and moist desquamation, and necrosis, using a 4-point Likert scale (0 = not at all; 1 = a little; 2 = quite a bit; 3 = very much) [32]. Finally, 1 trial also used a Skin Symptom Assessment (SSA) scale to record pruritus, pain/soreness, blistering/peeling, erythema, pigmentation, oedema, and trouble fitting brassieres [20].

**Fig. 2:**
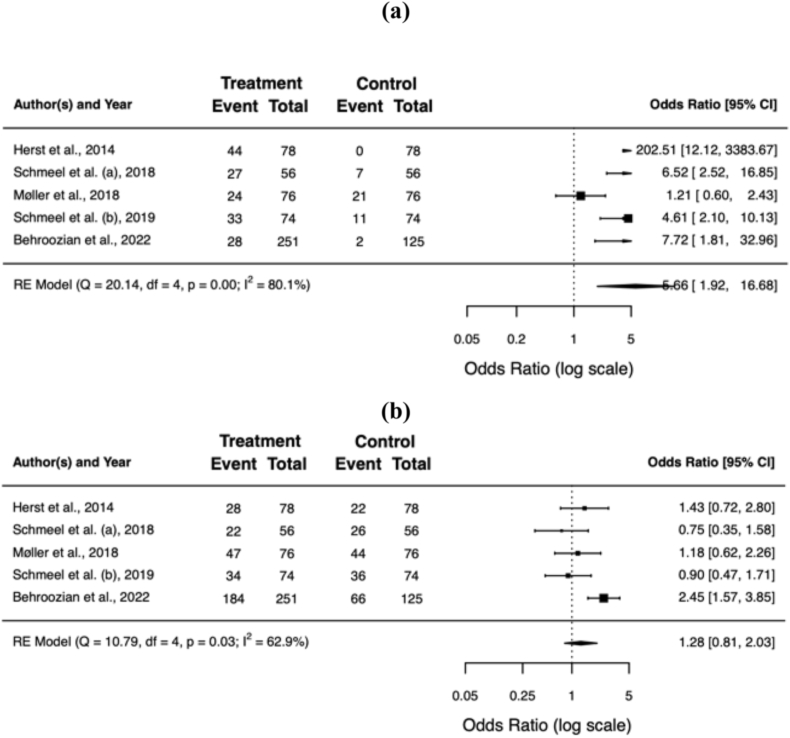

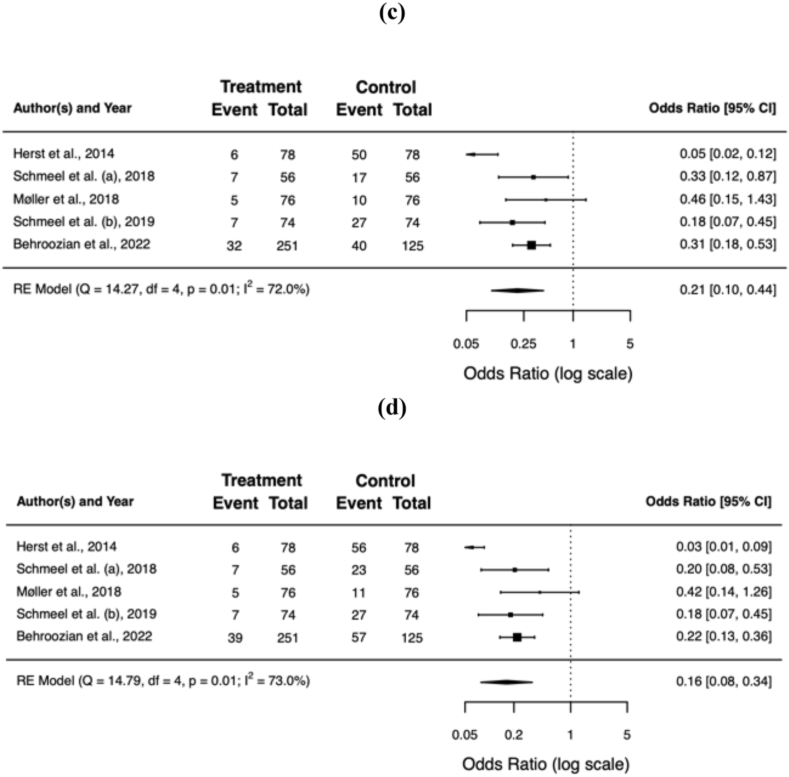

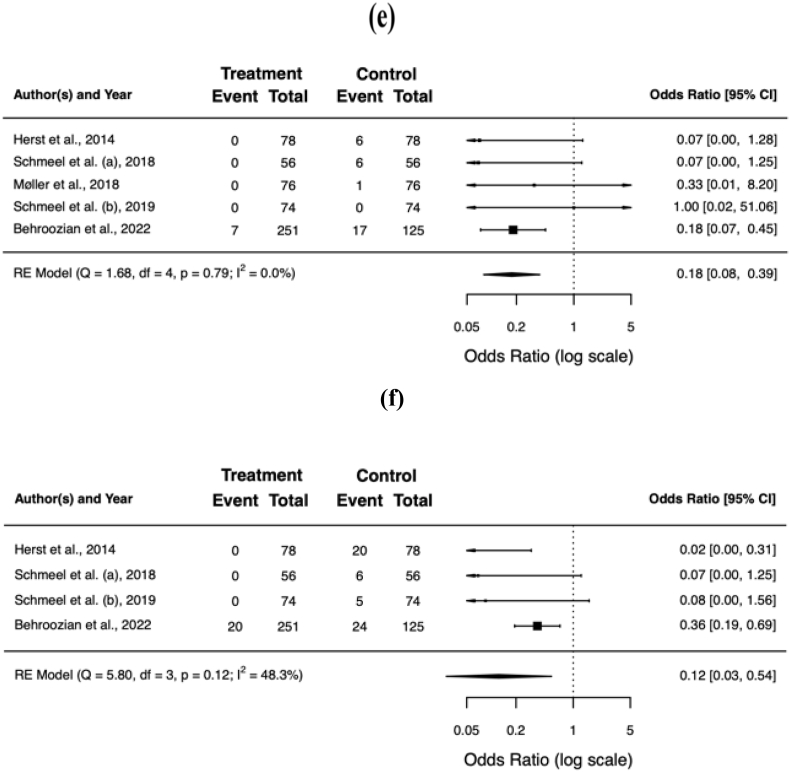
Forest plots of the individual and pooled effect sizes of developing grade 0 **(a)**, 1 **(b)**, 2 **(c)**, ≥2 **(d)**, or 3 **(e)** RD and moist desquamation **(f)**. Error bars indicate the 95% confidence interval (CI). RD = radiation dermatitis.

All but one of the included trials reported a significant difference in RD severity upon radiation treatment completion, favouring barrier film over standard skin care (Møller et al. found no significant difference in their blinded staff evaluation). A pooled and weighted analysis confirms this: 11% of patients developed grade ≥2 RD with barrier film, compared to 42% without (OR = 0.16; 95% CI 0.08−0.34; *p* < 0.001). The benefit of barrier films remained significant in patients developing grade 3 RD (1% vs. 7%; OR = 0.18; 95% CI 0.08−0.39; *p* < 0.001) and moist desquamation (2% vs. 16%; OR = 0.12; 95% CI 0.03−0.54; *p* = 0.006). Table 3 summarises the clinician-reported outcomes for the individual trials, Fig. 2a−f shows the forest plots of the individual and pooled effect sizes.

**Table 3:** Clinician-reported outcome.

authors	assessment tool	blinding	barrier film (%)^a^					control (%)^a^				
			G0	G1	G2	G3	MD	G0	G1	G2	G3	MD
Herst et al.	RTOG, RISRAS	none	56	36	8	0	0	0	28	64	8	26
Schmeel et al. (a)	RTOG	none	48	39	13	0	0	13	46	30	11	11
Møller et al.	RTOG	observer	31	62	7	0	N/A	28	58	13	1	N/A
Schmeel et al. (b)	CTCAE v4.03	none	45	46	10	0	0	15	49	37	0	7
Behroozian et al.	CTCAE v5.0, RISRAS, SSA	none	11	73	13	3	8	2	53	32	14	19
**total**			**38**°	**51**	**10**°	**1**°	**2**°	**11**	**47**	**35**	**7**	**16**

RTOG = Radiation Therapy Oncology Group; RISRAS = Radiation-Induced Skin Reaction Assessment Scale; CTCAE = Common Terminology Criteria for Adverse Events; SSA = Skin Symptom Assessment; G = grade; MD = moist desquamation; N/A = not available.

° *p* < 0.01.

a Radiation dermatitis assessment using RTOG/CTCAE.

### 3.4 Patient-reported outcome

Each of the included trials also assessed patient-reported outcome. The RISRAS contains an evaluation of patient-experienced pain, itching, burning, and subsequent limitations in daily activities, using a 4-point Likert scale (0 = not at all; 1 = a little; 2 = quite a bit; 3 = very much) [32]. Møller et al. used a patient-reported outcome questionnaire based on the RISRAS. Behroozian et al. also recorded the patient-assessed SSA (same items as clinicians).

**Fig. 3:**
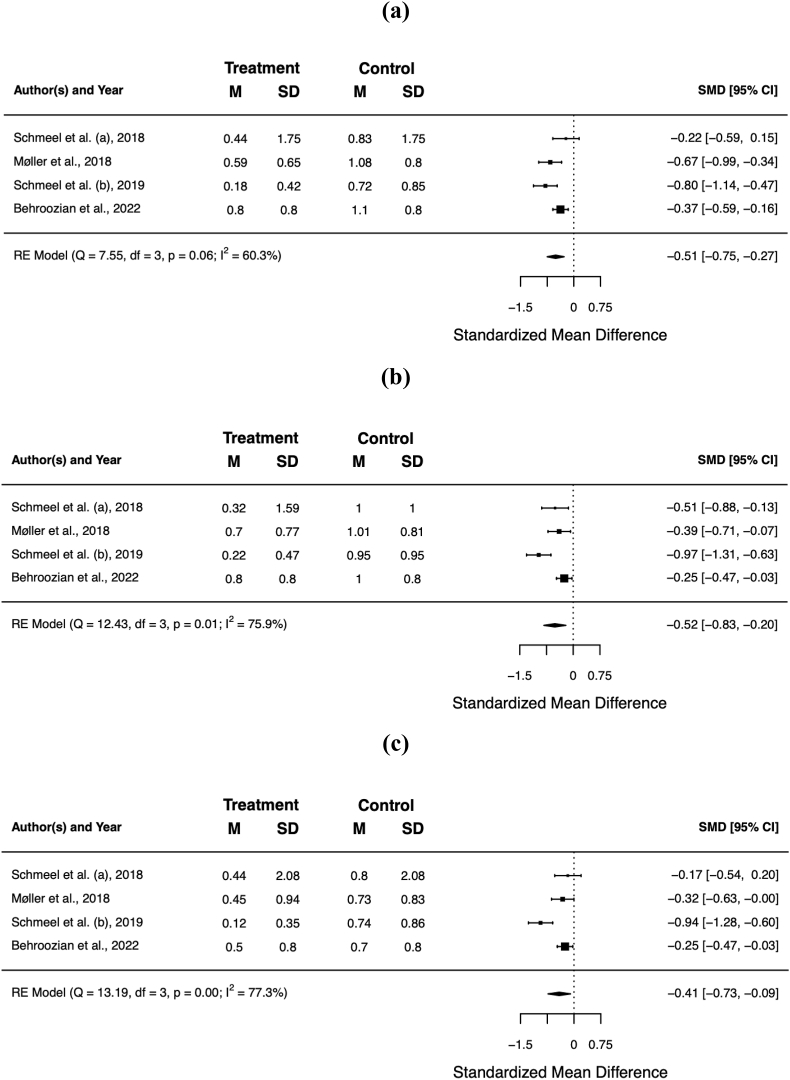

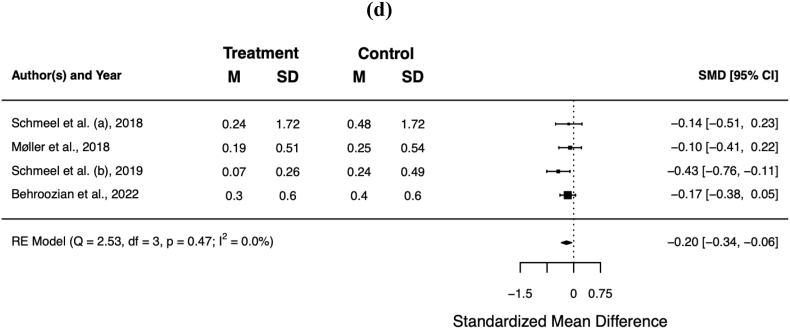
Forest plots of the mean item score for patient-reported pain **(a)**, itching **(b)**, burning **(c)**, and limitations in daily activities **(d)**. Error bars indicate the 95% confidence interval (CI).

Overall, barrier films resulted in a significant reduction of the mean item score for pain (standardised mean difference [SMD] −0.51; 95% CI −0.75, −0.27; *p* < 0.001), itching (SMD −0.52; 95% CI −0.83, −0.20; *p* = 0.001), burning (SMD −0.41; 95% CI −0.73, −0.09; *p* = 0.011), and limitations in daily activities (SMD −0.20; 95% CI −0.20, −0.34; *p* = 0.007). Table 4 summarises the patient-reported outcomes for the individual trials, Fig. 3a−d shows the respective forest plots.

**Table 4:** Patient-reported outcome. Scores in **bold** were statistically significant better for barrier film in the individual trials (*p* < 0.05).

authors	assessment tool	barrier film^b^				control^b^			
		pain	itching	burning	limitation	pain	itching	burning	limitation
Herst et al.	RISRAS	N/A^c^				N/A^c^			
Schmeel et al. (a)	RISRAS	**0.44**	**0.32**	0.44	0.24	0.83	1.00	0.80	0.48
Møller et al.	PROM^a^	**0.59**	**0.70**	**0.45**	0.19	1.08	1.01	0.73	0.25
Schmeel et al. (b)	RISRAS	**0.18**	**0.22**	**0.12**	**0.07**	0.72	0.95	0.74	0.24
Behroozian et al.	RISRAS, SSA	**0.80**	**0.80**	**0.50**	0.30	1.10	1.00	0.70	0.40
**total**		**0.66**°	**0.67**°	**0.44**°	**0.25**°	**1.02**	**1.00**	**0.72**	**0.37**

RISRAS = Radiation-Induced Skin Reaction Assessment Scale; PROM = patient-reported outcome measures; SSA = Skin Symptom Assessment; N/A = not available.

° *p* < 0.05.

a Based on RISRAS.

b Mean maximum scores.

c Herst et al. only reported the combined (clinician + patient) RISRAS scores.

### 3.5 Objective assessment

Two trials used an objective RD assessment method to corroborate their findings [18,19]: reflectance spectrophotometry is a validated tool to objectively assess skin colour, as it correlates with clinician-reported outcome [33]. Lower L* values describe darker skin (hyperpigmentation), whereas higher a* values are interpreted as an increased erythema intensity. Schmeel et al. performed 5 spectrophotometric readings in each of the breast compartments upon radiation treatment completion.

**Fig. 4:**
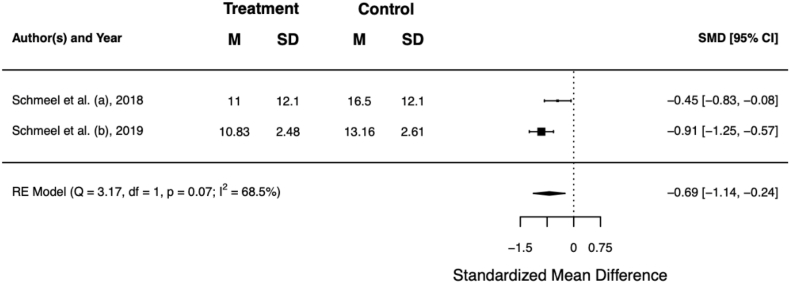
Forest plot of the mean a* value. Higher values of this objective assessment method are interpreted as an increased erythema intensity. Error bars indicate the 95% confidence interval (CI).

Overall, the mean a* value was significantly lower in the Hydrofilm compartments (SMD –0.69; 95% CI –1.14, −0.24; *p* = 0.003), which supports the beneficial effect of barrier film on clinician-reported RD severity. Table 5 summarises the outcome of the objective RD assessment, Fig. 4 shows the respective forest plot.

**Table 5:** Objective assessment of radiation dermatitis using reflectance spectrophotometry. Lower L* values describe darker skin (hyperpigmentation), whereas higher a* values are interpreted as an increased erythema intensity. Scores in **bold** were statistically significant better for barrier film in the individual trials (*p* < 0.05).

authors	barrier film		control	
	L*	a*	L*	a*
Schmeel et al. (a)	N/A	**11.00**	N/A	16.50
Schmeel et al. (b)	**65.31**	**10.83**	59.90	13.16
**total**	**N/A**	**10.90**°	**N/A**	**14.60**

N/A = not available.

° *p* < 0.01.

### 3.6 Topical corticosteroid use

**Fig. 5:**
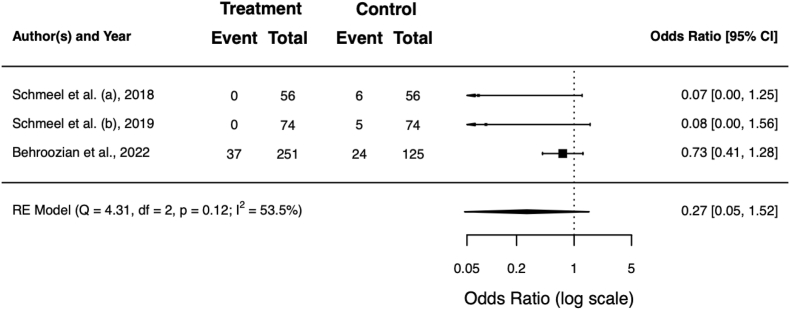
Forest plot of the topical corticosteroid prescription rate to alleviate RD-induced symptoms. RD = radiation dermatitis.

Three trials reported on the use of physician-prescribed topical corticosteroids to alleviate RD-induced symptoms such as pain or itching [[18], [19], [20]]. The pooled rate of patients receiving topical corticosteroids among these trials was 5% for patients or compartments with barrier film vs. 12% for the respective controls (OR = 0.27; 95% CI 0.05−1.52; *p* = 0.136). Table 6 shows the rates for the individual trials, Fig. 5 the respective forest plot.

**Table 6:** Rate of physician-prescribed topical corticosteroids to alleviate RD-induced symptoms such as pain or itching. Scores in **bold** were statistically significant better for barrier film in the individual trials (*p* < 0.05). Overall, the difference was not significant (*p* = 0.136).

authors	barrier film (%)	control (%)
Schmeel et al. (a)	**0**	11
Schmeel et al. (b)	**0**	7
Behroozian et al.	15	19
**total**	**5**	**12**

RD = radiation dermatitis.

Topical antibiotic use was documented by 1 trial only [20]. Here, a significant reduction was observed in the Mepitel film group (23% vs. 43%; *p* < 0.0001).

### 3.7 Tolerability, side effects, and patient experience

All trials reported on the tolerability and side effects of the barrier film used. As such, itching, rash, and the development of blisters under the applied barrier film were documented by each of the included trials, with a mean prevalence of 7%, 5%, and 2%, respectively. Most of these side effects were mild and self-limiting, rarely requiring any additional treatment (other than adjusting, changing, or removing the barrier film). Cutaneous side effects are mainly related to shear stress at the edges of the barrier film due to suboptimal film application. There seems to be a learning curve for healthcare providers regarding barrier film application: Schmeel et al. observed a reduced rate of side effects in their follow-up trial with Hydrofilm when compared to the initial trial performed at the same center [18,19].

Three trials assessed patient-reported experience measures. Overall, there was a high patient acceptance of barrier films. On average, 82% of patients preferred the barrier film over standard of care, while 87% would recommend its use to other patients to prevent RD. Table 7 shows an overview of the tolerability and patient experience.

**Table 7:** Tolerability, side effects, patient experience, and costs of barrier film use.

authors	side effects with barrier film (%)			experience (%)		costs^b^	
	itching	rash	blisters	preferred	recommend	number of films (*n*)	price (EUR)^c^
Herst et al.	4	0	0	92	N/A	10	71.48
Schmeel et al. (a)	16	8	7	N/A		6	20.00
Møller et al.	9^a^	9^a^	3	76	84	8	N/A
Schmeel et al. (b)	8	5	0	77	89	N/A	20.00
Behroozian et al.	0	1	0	N/A		21	62.73
**total**	**7**	**5**	**2**	**82**	**87**	**11**	**51.40**

N/A = not available.

a Møller et al. only reported the combined prevalence of itching and rash, which was 9%.

b Mean per patient to cover the treated breast/chest wall over the entire treatment course. The respective number of films and price for the first 4 trials (where only either the medial or lateral compartment was covered with barrier film) were doubled to estimate the actual number of films and costs for the entire breast/chest wall.

c Costs for raw material in the form of barrier film. Costs yielded by application of barrier film by a trained healthcare provider are not included.

### 3.8 Costs and time

All trials stated either the mean number of barrier films needed per patient over the entire treatment course, or the mean total raw material costs yielded by barrier film use. Behroozian et al. reported the highest number of barrier films per patient, since they included patients after lumpectomy only if they had a postoperative cup size ≥ C. Across all trials, an average of 11 barrier films was needed per patient. Hydrofilm costs were lower in comparison with Mepitel film.

Herst et al. reported an average of 5–10 min to replace a single barrier film, whereas Behroozian et al. calculated a mean time spent adjusting or replacing the barrier film of 51 min for the entire treatment period (55 min for lumpectomy patients and 46 min for mastectomy patients). None of the trials estimated the costs generated by the correct application of the barrier film by a trained healthcare provider. Table 7 shows an overview of the costs generated by barrier films across the individual trials.

## 4 Discussion

As the evidence on the use of barrier films for the prevention of acute RD in the context of adjuvant whole-breast or chest wall irradiation emerges, there is a need for a comprehensive appraisal of the available data. We conducted a systematic review of international literature and included 5 high-level RCTs in the quantitative meta-analysis (2 with Hydrofilm and 3 with Mepitel film), to assess clinician- and patient-reported benefits of barrier film usage as well as its safety and cost-effectiveness.

Four trials used an intrapatient control receiving standard of care. Apart from the assumed positive impact on patient recruitment (all patients receive the intervention), each patient acts as their own control, which minimises confounding and omits the need for stratification based on factors known to influence the RD risk (e.g. breast size, skin type, use of a bolus, fractionation regimen). Only Behroozian et al. had to stratify patients accordingly, since they recruited a physical control group receiving only standard of care. In the latter trial, there were no significant differences in patient characteristics between the two groups. Lighter skin tones were, however, more common in the group receiving Mepitel film (33% vs. 22%; *p* = 0.056), which is a known protective factor for RD development and could have slightly skewed the results in favour of the intervention [4]. In addition, this trial only included patients at higher risk of developing RD (i.e. patients after lumpectomy with a large postoperative breast size and patients after mastectomy) to maximise the effect size and assess the benefit of Mepitel film in this high-risk subgroup [4,6]. As the number of patients contributed by Behroozian et al. constitutes about 57% of the pooled sample size in this meta-analysis, there might be a slight overestimation of the overall effect size of barrier films.

A further limitation of these RCTs is the overall absence of clinician and/or patient blinding, due to the visible nature of the intervention. Møller et al. were the only trial that implemented a blinded skin assessment (of the bare skin, i.e. after removal of the barrier film) by a trained physician upon radiation treatment completion. Overall, they found no significant difference in RD severity between the medial and lateral breast compartment (*p* = 0.100). Only in the small subgroups of patients undergoing chest wall irradiation (*n* = 16) and conventional fractionation (*n* = 20), there was a significant difference favouring barrier film use (*p* = 0.005 and *p* = 0.002, respectively). This might be related to the intrinsically increased RD risk (both incidence and severity) in these patient populations [4,6]. The primary endpoint of Møller et al., i.e. patient-reported symptoms and experience, was however met.

The lack of blinding might have introduced observational bias in the respective clinician- and patient-reported outcome assessments and is regarded as the main source of bias in these RCTs and the subsequent meta-analysis (Supplement 1). The use of validated non-invasive objective assessment methods (such as reflectance spectrophotometry) might overcome this and should thus be considered in future trials in this context [33,34]. In the case of Schmeel et al., the spectrophotometric readings supported the clinician-reported differences in erythema intensity in favour of barrier film.

Of great added value are the patient-reported outcomes in each of the trials included in this meta-analysis, especially since symptoms caused by RD tend to be significantly underreported by clinicians: Behroozian et al. previously investigated these discrepancies in 777 patients undergoing adjuvant whole-breast or chest wall irradiation and found only low to moderate concordance between patients and clinicians [10]. The authors thus conclude that clinician-reported outcomes alone are insufficient to assess RD (or the subsequent effect of an intervention), as they do not adequately take the impact on a patient's quality of life into account. The RISRAS, which has both a clinician and patient component, is validated and easy to implement in this context [32]. Its use should therefore also be considered in future trials.

Another limitation of the included RCTs is the limited information on treatment techniques such as three-dimensional conformal radiation therapy (3D-CRT), IMRT, or VMAT, and the subsequent absence of stratification based on it. VMAT has been shown to result in significantly lower dermatitis grades when compared to IMRT [4]. Furthermore, the majority (>90%) of patients included in this meta-analysis were of Caucasian descent and had lighter Fitzpatrick skin types. It is therefore difficult to assess the potential benefits of barrier film in the smaller subgroup of patients with darker skin complexion, a known risk factor for the development of RD [4]. Future trials should aim to assess the respective risk reductions in each of these subgroups.

The overall benefit of barrier films as a prophylactic measure in the context of whole-breast and chest wall irradiation is apparent: improved clinician- and patient-reported outcomes, confirmed by objective measurements, as well as an overall favourable patient experience and cost-benefit ratio make for an excellent tool in the prevention of acute RD, especially in patients with high risk of developing it. The possible side effects of barrier film application such as itching or rash are mild and mostly self-limiting. Care should however be taken to ensure proper tension-free placement on the skin. If multiple barrier films are required to cover the irradiated area (e.g. in patients with large breast size), overlapping of different films should be avoided to prevent increased dose build-up. The negligible bolus effect of a single barrier film implies that they can safely remain on the skin during the entire radiation treatment, which promotes patient comfort.

Challenges with barrier films include difficult application and poor adherence to regions with increased perspiration and friction, such as the axillary and clavicular region in patients undergoing locoregional irradiation – of all areas, these in particular tend to have a predilection for the development of acute RD and moist desquamation [8]. In these patients, more frequent replacement of the barrier film might be necessary, thereby increasing total treatment time and costs. Patients with large breasts also require more frequent adjustments (e.g. due to rolling up of the barrier film at the edges) and replacements of their barrier film for this very reason. Furthermore, care should be taken to prevent distortion of the breast shape, which could otherwise interfere with the radiation treatment setup (Schmeel et al. and Behroozian et al. used weekly cone beam computed tomography to assure correct film application and positioning). Film application and (daily) position control should ideally be carried out by an experienced healthcare provider, which is time-consuming and yields additional costs. The possible interference of barrier films with treatment verification through surface-guided radiotherapy has not been studied so far and should be the aim of future trials.

The use of barrier films as a prophylactic RD measure has also been investigated in the context of head and neck irradiation, but data are sparse. Wooding et al. (*n* = 33) investigated the use of Mepitel film in a randomised intrapatient-controlled trial and found a 27–29% reduction of the combined RISRAS, as well as a 28–37% reduction of the moist desquamation rate (in a New Zealand and a Chinese cohort, respectively) [35]. These results were confirmed by Yan et al. (*n* = 39): with a similar study design, they found respective reductions of 30% and 41% [29]. In the latter trial, 80% of patients preferred Mepitel film over standard of care skin cream. Both trials, however, ran into the same obstacles: the complex anatomy of the head and neck region resulted in poor adherence of the Mepitel film to the skin (especially in patients with stubble or beard), which led to itching and discomfort. For this very reason, the RAREST-01 trial, which sought to further corroborate the use of Mepitel film for RD prevention in head and neck irradiation, was stopped prematurely (46% of patients could not tolerate Mepitel film) [36].

Some systematic reviews on the use of barrier films for RD prevention have been conducted in the past [[37], [38], [39], [40], [41]]. Often, however, there were considerable methodological differences between the included studies, e.g. regarding the nature of the barrier film, irradiated area, skin care control, study design, endpoints, and assessment methods. This lack of standardisation between included studies generally resulted in inconsistent and weak recommendations [12]. A quite comprehensive overview was recently published, on the basis of which Hydrofilm and Mepitel film are recommended for RD prevention by the Multinational Association of Supportive Care in Cancer (MASCC) [41,42]. The latter, however, also investigated the use of barrier creams and film-forming gels, in different oncological contexts, and included trials published before September 2020 only, meaning that the most comprehensive barrier film RCT to date was not included [20]. In the current systematic review and meta-analysis, we focussed on RD prevention with two comparable barrier films in the context of whole-breast and chest wall irradiation and only included RCTs with similar skin care controls and quantitatively comparable outcomes. Additionally, we comprehensively evaluated the bolus effect, side effects, patient experience, and cost-benefit ratio, which were not considered in previous reports. Although Hydrofilm and Mepitel film possess some different properties (mainly in terms of the adhesive used), they are applied in the same way and have an identical mechanism of action by creating a mechanical barrier and were, therefore, included in a single meta-analysis. The decision on when to use which barrier film should be based on patient characteristics. Hydrofilm has stronger adhesive potential and could be preferred in complex anatomical regions (e.g. women with larger breasts, skin folds), very active patients, or those who tend to experience more excessive perspiration. Mepitel film, in contrast, is easier to remove and might be preferred in patients with more sensitive or brittle skin. Patient allergies, pricing, clinician preference and experience might also influence the choice of barrier film. Barrier creams and film-forming gels (e.g. StrataXRT® or 3 M Cavilon No Sting Barrier Film®) were explicitly excluded from the analysis as the evidence in the context of breast irradiation is currently limited and these products exhibit substantial differences when compared to regular barrier films, which would have increased the heterogeneity of the included trials and results.

Other promising topical preventive measures in the form of creams or medical devices are currently being investigated in the context of whole-breast and chest wall irradiation [[43], [44], [45]]. Furthermore, advances in patient positioning and radiation treatment planning and delivery (e.g. ultrahypofractionation, partial-breast irradiation) are a valuable approach towards reducing RD burden [46]. The effects of barrier film application should also be explored in these contexts, also focussing on dosimetric aspects of radiation treatment.

## 5 Conclusion

Barrier films for acute RD prevention improve clinician- and patient-reported outcomes in the context of adjuvant whole-breast or chest wall irradiation, which in turn improves quality of life. Results were consistent across all RCTs included in this meta-analysis, despite certain possible bias due to methodological differences and the general absence of blinding. The use of barrier films should therefore be routinely considered, especially in patients with high risk of developing RD.

## Author contributions

All authors contributed to the study conception and design. Material preparation, data collection and analysis were performed by C.S.D., E.D., and L.C.S. The first draft of the manuscript was written by C.S.D. and reviewed and edited by L.C.S. All authors commented on previous versions of the manuscript. All authors read and approved the final manuscript.

## Funding and conflict of interest

The authors declare that no funds, grants, or other support were received during the preparation of this manuscript. There are no relevant financial or non-financial interests to disclose, nor are there any proprietary interests in the material discussed in this article.

## Ethics statement

This systematic review of literature and meta-analysis required no ethical approval.

## References

[1] Shaitelman S.F., Schlembach P.J., Arzu I., Ballo M., Bloom E.S., Buchholz D. (2015). Acute and short-term toxic effects of conventionally fractionated vs. hypofractionated whole-breast radiation: a randomized clinical trial. JAMA Oncol.

[2] Schmeel L.C., Koch D., Schmeel F.C., Röhner F., Schoroth F., Bücheler B.M. (2020). Acute radiation-induced skin toxicity in hypofractionated vs. conventional whole-breast irradiation: an objective, randomized multicenter assessment using spectrophotometry. Radiother Oncol J Eur Soc Ther Radiol Oncol.

[3] Parker J.J., Rademaker A., Donnelly E.D., Choi J.N. (2017). Risk factors for the development of acute radiation dermatitis in breast cancer patients. Int J Radiat Oncol Biol Phys.

[4] Böhner A.M.C., Koch D., Schmeel F.C., Röhner F., Schoroth F., Sarria G.R. (2020). Objective evaluation of risk factors for radiation dermatitis in whole-breast irradiation using the spectrophotometric L*a*b* colour-space. Cancers.

[5] Behroozian T., Milton L.T., Shear N.H., McKenzie E., Razvi Y., Karam I. (2021). Radiation dermatitis assessment tools used in breast cancer: a systematic review of measurement properties. Support Care Cancer.

[6] Xie Y., Wang Q., Hu T., Chen R., Wang J., Chang H. (2021). Risk factors related to acute radiation dermatitis in breast cancer patients after radiotherapy: a systematic review and meta-analysis. Front Oncol.

[7] Haruna F., Lipsett A., Marignol L. (2017). Topical management of acute radiation dermatitis in breast cancer patients: a systematic review and meta-analysis. Anticancer Res.

[8] Singh M., Alavi A., Wong R., Akita S. (2016). Radiodermatitis: a review of our current understanding. Am J Clin Dermatol.

[9] Rzepecki A.K., Birnbaum M., Fox J.L., Kabarriti R., Garg M.K., Daily J. (2018). Characterizing the effects of radiation dermatitis on quality of life. Int J Radiat Oncol Biol Phys.

[10] Behroozian T., Milton L., Zhang L., Lou J., Karam I., Lam E. (2021). How do patient-reported outcomes compare with clinician assessments? A prospective study of radiation dermatitis in breast cancer. Radiother Oncol J Eur Soc Ther Radiol Oncol.

[11] Barnes L., Kaya G., Rollason V. (2015). Topical corticosteroid-induced skin atrophy: a comprehensive review. Drug Saf.

[12] Finkelstein S., Kanee L., Behroozian T., Wolf J.R., van den Hurk C., Chow E. (2022). Comparison of clinical practice guidelines on radiation dermatitis: a narrative review. Support Care Cancer.

[13] Layer K., Layer J.P., Glasmacher A.R., Sarria G.R., Böhner A.M.C., Layer Y.L. (2023). Risk assessment, surveillance, and nonpharmaceutical prevention of acute radiation dermatitis: results of a multicentric survey among the German-speaking radiation oncology community. Strahlenther Onkol.

[14] Palfreyman S., Stevens J. (2010). Use of Hydrofilm and Hydrofilm plus in the community: an assessment. Br J Community Nurs.

[15] Diggelmann K.V., Zytkovicz A.E., Tuaine J.M., Bennett N.C., Kelly L.E., Herst P.M. (2010). Mepilex Lite dressings for the management of radiation-induced erythema: a systematic inpatient controlled clinical trial. Br J Radiol.

[16] Herst P.M. (2014). Protecting the radiation-damaged skin from friction: a mini review. J Med Radiat Sci.

[17] Herst P.M., Bennett N.C., Sutherland A.E., Peszynski R.I., Paterson D.B., Jasperse M.L. (2014). Prophylactic use of Mepitel Film prevents radiation-induced moist desquamation in an intra-patient randomised controlled clinical trial of 78 breast cancer patients. Radiother Oncol.

[18] Schmeel L.C., Koch D., Stumpf S., Leitzen C., Simon B., Schüller H. (2018). Prophylactically applied Hydrofilm polyurethane film dressings reduce radiation dermatitis in adjuvant radiation therapy of breast cancer patients. Acta Oncol (Madr).

[19] Schmeel L.C., Koch D., Schmeel F.C., Bücheler B., Leitzen C., Mahlmann B. (2019). Hydrofilm polyurethane films reduce radiation dermatitis severity in hypofractionated whole-breast irradiation: an objective, intrapatient randomized dual-center assessment. Polymers (Basel).

[20] Behroozian T., Milton L., Karam I., Zhang L., Ding K., Lou J. (2022). Mepitel film for the prevention of acute radiation dermatitis in breast cancer: a randomized multicenter open-label phase III trial. J Clin Oncol.

[21] Paterson D.B., Poonam P., Bennett N.C., Peszynski R.I., Van Beekhuizen M.J., Jasperse M.L. (2012). Randomized intra-patient controlled trial of mepilex lite dressings versus aqueous cream in managing radiation-induced skin reactions post-mastectomy. J Cancer Sci Ther.

[22] Sterne J.A.C., Savović J., Page M.J., Elbers R.G., Blencowe N.S., Boutron I. (2019). RoB 2: a revised tool for assessing risk of bias in randomised trials. BMJ.

[23] Page M.J., McKenzie J.E., Bossuyt P.M., Boutron I., Hoffmann T.C., Mulrow C.D. (2021). The PRISMA 2020 statement: an updated guideline for reporting systematic reviews. BMJ.

[24] DerSimonian R., Laird N. (1986). Meta-analysis in clinical trials. Contr Clin Trials.

[25] Higgins J.P.T., Thompson S.G. (2002). Quantifying heterogeneity in a meta-analysis. Stat Med.

[26] Møller P.K., Olling K., Berg M., Habæk I., Haislund B., Iversen A.-M. (2018). Breast cancer patients report reduced sensitivity and pain using a barrier film during radiotherapy – a Danish intra-patient randomized multicentre study. Tech Innov Patient Support Radiat Oncol.

[27] Yee C., Wang K., Asthana R., Drost L., Lam H., Lee J. (2018). Radiation-induced skin toxicity in breast cancer patients: a systematic review of randomized trials. Clin Breast Cancer.

[28] Ben Amor R., Bohli M., Naimi Z., Aissaoui D., Mejri N., Yahyaoui J. (2023). Hypofractionated radiotherapy after breast-conserving surgery: clinical and dosimetric factors predictive of acute skin toxicity. Strahlenther Onkol.

[29] Yan J., Yuan L., Wang J., Li S., Yao M., Wang K. (2020). Mepitel Film is superior to Biafine cream in managing acute radiation-induced skin reactions in head and neck cancer patients: a randomised intra-patient controlled clinical trial. J Med Radiat Sci.

[30] Cox J.D., Stetz J., Pajak T.F. (1995). Toxicity criteria of the radiation therapy oncology group (RTOG) and the European organization for research and treatment of cancer (EORTC). Int J Radiat Oncol Biol Phys.

[31] Common Terminology Criteria for Adverse Events (CTCAE) Version 5.0 n.d. https://ctep.cancer.gov/protocoldevelopment/electronic_applications/docs/ctcae_v5_quick_reference_5x7.pdf.

[32] Noble-Adams R. (1999). Radiation-induced skin reactions. 3: evaluating the RISRAS. Br J Nurs.

[33] Momm F., Bartelt S., Haigis K., Grosse-Sender A., Witucki G. (2005). Spectrophotometric skin measurements correlate with EORTC/RTOG-common toxicity criteria. Strahlenther Onkol.

[34] Wengström Y., Forsberg C., Näslund I., Bergh J. (2004). Quantitative assessment of skin erythema due to radiotherapy - evaluation of different measurements. Radiother Oncol J Eur Soc Ther Radiol Oncol Eur Soc Ther Radiol Oncol.

[35] Wooding H., Yan J., Yuan L., Chyou T.-Y., Gao S., Ward I. (2018). The effect of Mepitel Film on acute radiation-induced skin reactions in head and neck cancer patients: a feasibility study. Br J Radiol.

[36] Rades D., Narvaez C.A., Splettstößer L., Dömer C., Setter C., Idel C. (2019). A randomized trial (RAREST-01) comparing Mepitel® Film and standard care for prevention of radiation dermatitis in patients irradiated for locally advanced squamous cell carcinoma of the head-and-neck (SCCHN). Radiother Oncol J Eur Soc Ther Radiol Oncol.

[37] Chan R.J., Webster J., Chung B., Marquart L., Ahmed M., Garantziotis S. (2014). Prevention and treatment of acute radiation-induced skin reactions: a systematic review and meta-analysis of randomized controlled trials. BMC Cancer.

[38] Wan B.A., Chan S., Herst P., Yee C., Popovic M., Lee J. (2019). Mepitel Film and Mepilex Lite for the prophylaxis and treatment of skin toxicities from breast radiation. Breast.

[39] Ginex P.K., Backler C., Croson E., Horrell L.N., Moriarty K.A., Maloney C. (2020). Radiodermatitis in patients with cancer: systematic review and meta-analysis. Oncol Nurs Forum.

[40] Fernández-Castro M., Martín-Gil B., Peña-García I., López-Vallecillo M., García-Puig M.E. (2017). Effectiveness of semi-permeable dressings to treat radiation-induced skin reactions. A systematic review. Eur J Cancer Care.

[41] Robijns J., Aquilano M., Banerjee S., Caini S., Wolf J.R., van den Hurk C. (2023). Barrier films and dressings for the prevention of acute radiation dermatitis: a systematic review and meta-analysis. Support Care Cancer.

[42] Behroozian T., Bonomo P., Patel P., Kanee L., Finkelstein S., van den Hurk C. (2023). Multinational Association of Supportive Care in Cancer (MASCC) clinical practice guidelines for the prevention and management of acute radiation dermatitis: international Delphi consensus-based recommendations. Lancet Oncol.

[43] Zhao H., Zhu W., Zhao X., Li X., Zhou Z., Zheng M. (2022). Efficacy of epigallocatechin-3-gallate in preventing dermatitis in patients with breast cancer receiving postoperative radiotherapy: a double-blind, placebo-controlled, phase 2 randomized clinical trial. JAMA Dermatol.

[44] Robijns J., Lodewijckx J., Puts S., Vanmechelen S., Van Bever L., Claes S. (2022). Photobiomodulation therapy for the prevention of acute radiation dermatitis in breast cancer patients undergoing hypofractioned whole-breast irradiation (LABRA trial). Laser Surg Med.

[45] Dejonckheere C.S., Böhner A.M.C., Schmitz E., Holderried T.A.W., Schmeel L.C., Brossart P. (2022). Non-invasive physical plasma for preventing radiation dermatitis in breast cancer: a first-in-human feasibility study. Pharmaceutics.

[46] Vesprini D., Davidson M., Bosnic S., Truong P., Vallieres I., Fenkell L. (2022). Effect of supine vs prone breast radiotherapy on acute toxic effects of the skin among women with large breast size: a randomized clinical trial. JAMA Oncol.

