# Supplementary material for: Barrier films for the prevention of acute radiation dermatitis in breast cancer: A systematic review and meta-analysis of randomised controlled trials
Source: Breast. 2023 Jul 5;71:31–41. doi: 10.1016/j.breast.2023.07.001 (PMC10404536; doi:10.1016/j.breast.2023.07.001)

**SUPPLEMENT**

**Supplement 1** Traffic light plot assessing the risk of bias for the included trials. D1 = random sequence generation (selection bias); D2 = allocation concealment (selection bias); D3 = blinding of participants and personnel (performance bias); D4 = blinding of outcome assessment (detection bias); D5 = incomplete outcome data (attrition bias); D6 = selective reporting (reporting bias); D7 = other bias.


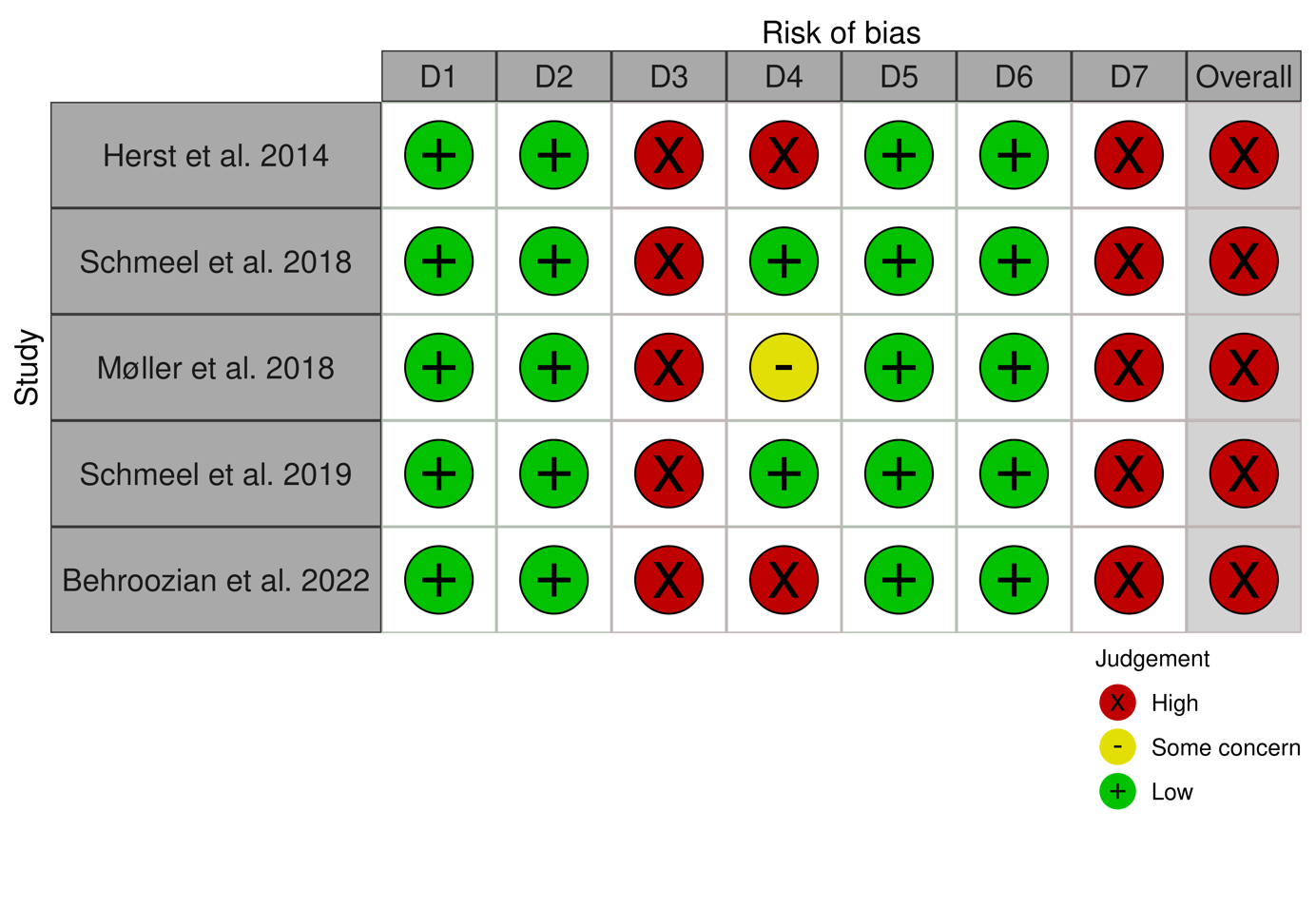

Supplement: Multimedia component 1 [file mmc1.docx]
